# Data supporting the cover crops benefits related to soil functionality in a 10-year cropping system

**DOI:** 10.1016/j.dib.2018.04.029

**Published:** 2018-04-18

**Authors:** Irene García-González, Chiquinquirá Hontoria, José Luis Gabriel, María Alonso-Ayuso, Miguel Quemada

**Affiliations:** a Departamento de Producción Agraria, Universidad Politécnica de Madrid, Avda. Complutense s/n., 28040 Madrid, Spain; b Instituto Nacional de Investigación y Tecnología Agraria y Alimentaria, Ctra. de la Coruña km. 7.5, 28040 Madrid, Spain

## Abstract

In this data article we provide different field parameters of an agricultural irrigated system under Mediterranean conditions. These parameters represent the response of variables related to soil functionality to different cover crops. Soil and plant samples were taken from fallow and cover crops treatments over the course of 10 years, with most variables measured every other year. This ample database provides reliable information to design sustainable agricultural practices under Mediterranean conditions. Researchers, policy makers and farmers are interested in the final outcome of this dataset. The data are associated with the research article entitled “Cover crops to mitigate soil degradation and enhance soil functionality in irrigated land” (García-González et al., 2018) [1].

**Specifications Table**

**Table:** 

Subject area	Agriculture- Soil Science.
More specific subject area	Restoring soil degradation by cover cropping.
Type of data	Tables and Excel files.
How data was acquired	Plant and soil samples were collected in a field experiment and analyzed in a laboratory. In addition, some measurements were taken directly in the field.
Data format	Raw and analyzed data.
Experimental factors	Samples were collected at different depths and years. In the laboratory, they were processed differently depending on the analysis.
Experimental features	A 10-year crop rotation (2006–2016) in which winter fallow was replaced by a grass and a legume cover crop.
Data source location	Aranjuez, Spain (40°03′N, 03°31′W, 550 m a.s.l.)
Data accessibility	Data are supplied with this article.

**Value of the data**

• Data from a long-term field experiment with cover crops will be useful to analyze the response to soil restoration after detrimental agricultural practices.
• Temporal evolution of carbon and nitrogen soil sequestration could serve as a benchmark in Mediterranean soils.
• This database provides relevant information about nitrogen movement down to 4 m.
• This complete database can be used in the development of more sustainable agricultural practices under the specific weather conditions of semi-arid regions.

## 1 Data

The parameters were obtained after collecting plant and soil samples in a field experiment during a period of 10 years. All field replications of crop biomass, C and N content, particulate organic matter (POM), water-stable aggregates (WSA) and infiltration rates are reported in Excel files (Appendix A). Crop biomass, cumulative C and N in the crop biomass, atmospheric N_2_ fixed by the vetch were measured in main crops (October sampling) and cover crops (March sampling) in the different treatments every year (Table 1, Table 2).

**Table 1:** Temporal evolution of the cumulative biomass produced by main summer crops (maize or sunflower) or cover crops (barley or vetch) and carbon input in the cover crops for the various treatments.

Year	Sampling	Cumulative biomass (Mg dm ha^−1^)			Cumulative C input (Mg C ha^−1^)		
		Fallow	Barley	Vetch	Fallow	Barley	Vetch
2006	October	0	0	0	0.42	0.42	0.42
2007	March	0	6.24	5.06	0.84	2.97	2.46
2007	October	24.68	26.34	26.25	1.26	3.39	2.85
2008	March	24.68	28.59	26.83	1.26	4.27	3.07
2008	October	48.77	49.89	48.97	1.68	4.69	3.49
2009	March	48.77	51.72	50.31	1.69	5.43	4.04
2009	October	68.89	71.17	71.67	2.10	5.83	4.45
2010	March	68.89	75.28	73.65	2.10	7.36	5.06
2010	October	85.49	92.45	90.61	2.52	7.77	5.47
2011	March	85.49	94.85	91.31	2.54	8.74	5.75
2011	October	85.49	94.85	91.31	2.54	8.74	5.75
2012	March	85.49	97.98	92.53	2.55	10.01	6.23
2012	October	85.49	97.98	92.53	2.59	10.05	6.27
2013	March	85.49	101.4	95.12	2.59	11.50	7.38
2013	October	102.9	111.3	118.4	3.01	11.92	7.79
2014	March	102.9	111.8	119.5	3.01	12.14	8.23
2014	October	117.9	127.6	136.8	3.44	12.57	8.66
2015	March	117.9	131.3	140.9	3.44	14.14	10.36
2015	October	121.4	133.9	144.9	3.48	14.18	10.40
2016	March	121.4	135.8	146.1	3.48	15.00	10.92
2016	October	132.5	149.7	160.5	3.91	15.43	11.35

**Table 2:** Temporal evolution of the cumulative nitrogen in the cover crops.

		Cumulated N in cover crops (kg N ha^−^^1^)		
		Fixed by vetch	Barley	Vetch
2007	March	121.2	156.9	179.3
2008	March	127.6	196.0	199.4
2009	March	152.9	235.1	255.0
2010	March	199.6	312.4	310.4
2011	March	215.0	350.7	333.3
2012	March	246.5	400.8	373.6
2013	March	326.9	441.1	470.1
2014	March	364.9	452.1	511.4
2015	March	486.9	484.9	634.4
2016	March	519.5	508.3	668.7

Every two years after harvesting main crops (maize or sunflower), soil organic carbon (SOC) and soil organic nitrogen (SON) were determined at two different depths, 0–5 cm and 5–20 cm (Table 3). In addition, SOC was obtained at the beginning and at the end of the field experiment until 1 m depth (Table 4). In parallel analyses, POM and WSA were obtained every two years after main crops (Table 5). Infiltration rate was measured in the field in three years (Table 6). At the end of the field experiment (November 2016), soil inorganic N content was obtained in several layers until 4 m depth in barley and fallow treatments (Table 7).

**Table 3:** Temporal evolution of soil organic carbon (SOC) and soil organic nitrogen (SON), expressed either as concentration (%) or content (Mg ha^−1^) at two different depths.

Year	Treatment	SOC (%)		SOC (Mg ha^−^^1^)		SON (%)		SON (Mg ha^−1^)	
		0–5 cm	5–20 cm	0–5 cm	5–20 cm	0–5 cm	5–20 cm	0–5 cm	5–20 cm
2006	Fallow	1.05	1.00	7.36	6.94	0.12	0.12	0.85	0.83
2008	Fallow	1.02	0.97	7.32	6.57	0.14	0.12	0.93	0.79
2008	Barley	1.23	0.96	8.43	6.52	0.14	0.12	0.96	0.82
2008	Vetch	1.13	0.92	7.76	6.26	0.14	0.12	0.97	0.81
2010	Fallow	1.15	0.83	8.49	6.46	0.13	0.12	0.93	0.91
2010	Barley	1.38	0.86	10.18	6.74	0.14	0.12	1.03	0.91
2010	Vetch	1.36	0.79	10.06	6.14	0.14	0.11	1.03	0.87
2012	Fallow	1.23	0.91	8.12	7.20	0.14	0.10	0.92	0.82
2012	Barley	1.49	0.96	9.81	7.59	0.15	0.11	0.99	0.83
2012	Vetch	1.44	0.90	9.47	7.05	0.16	0.11	1.03	0.87
2014	Fallow	1.35	0.88	8.98	6.93	0.15	0.10	0.98	0.78
2014	Barley	1.58	0.96	10.49	7.49	0.17	0.10	1.13	0.80
2014	Vetch	1.54	0.93	10.22	7.32	0.17	0.10	1.12	0.78
2016	Fallow	1.37	0.89	9.14	6.95	0.16	0.12	1.07	0.94
2016	Barley	1.62	0.94	10.83	7.35	0.17	0.12	1.16	0.92
2016	Vetch	1.60	0.93	10.69	7.25	0.18	0.12	1.19	0.97

**Table 4:** Soil organic carbon (SOC) distribution according to soil depth at the beginning of the experiment (2006) and at the end (2016) for the various treatments.

Year	Soil depth (cm)	SOC (%)		
		Fallow	Barley	Vetch
2006	0–5	1.05	–	–
2006	5–20	1.00	–	–
2006	20–40	0.81	–	–
2006	40–60	0.50	–	–
2006	60–80	0.43	–	–
2006	80–100	0.35	–	–
2016	0–5	1.37	1.22	1.60
2016	5–20	0.89	0.94	0.93
2016	20–40	0.79	0.81	0.72
2016	40–60	0.60	0.50	0.55
2016	60–80	0.41	0.41	0.48
2016	80–100	0.38	0.39	0.41

**Table 5:** Temporal evolution of particulate organic matter (POM) and water-stable aggregates (WSA) for the various treatments at two different depths.

Year	Treatment	POM (%)		WSA (%)	
		0–5 cm	5–20 cm	0–5 cm	5–20 cm
2006	Fallow	0.24	0.22	26.98	26.69
2008	Fallow	0.29	0.20	25.33	20.48
2008	Barley	0.36	0.21	35.24	27.18
2008	Vetch	0.31	0.18	28.46	22.05
2010	Fallow	0.29	0.14	34.54	30.79
2010	Barley	0.37	0.15	56.18	30.95
2010	Vetch	0.40	0.13	44.99	25.38
2012	Fallow	0.33	0.12	37.63	18.18
2012	Barley	0.44	0.16	55.02	21.87
2012	Vetch	0.44	0.16	47.79	15.35
2014	Fallow	0.35	0.15	43.30	32.13
2014	Barley	0.47	0.18	68.07	37.12
2014	Vetch	0.44	0.16	53.47	39.49
2016	Fallow	0.36	0.13	39.62	33.24
2016	Barley	0.41	0.12	61.23	44.44
2016	Vetch	0.45	0.13	48.05	33.87

**Table 6:** Temporal evolution of the infiltration rates for the various treatments.

Year	Treatment	Infiltration (cm h^−1^)
2010	Fallow	0.49
2010	Barley	1.33
2010	Vetch	1.21
2012	Fallow	3.21
2012	Barley	17.53
2012	Vetch	5.96
2016	Fallow	9.70
2016	Barley	23.57
2016	Vetch	15.07

**Table 7:** Soil inorganic N content distribution according to depth at the end of the experiment (2016) for the barley and fallow treatments.

Soil depth (m)	Soil inorganic N content (kg N ha^−1^)	
	Fallow	Barley
0–0.2	4.40	9.63
0.2–0.4	5.49	3.87
0.4–0.6	1.99	1.45
0.6–0.8	1.03	0.67
0.8–1	3.83	1.49
1–1.33	6.71	2.52
1.33–1.67	6.95	2.60
1.67–2	4.61	2.19
2–2.33	5.29	2.69
2.33–2.67	10.19	5.47
2.67–3	12.83	6.98
3–3.33	11.19	5.62
3.33–3.67	15.50	6.87
3.67–4	10.22	3.15

## 2 Experimental design, materials and methods

### 2.1 The experimental site and design

The field experiment was conducted at La Chimenea Field Station (40°03′N, 03°31′W, 550 m a.s.l.) in the central Tajo river basin near Aranjuez (Madrid, Spain) from April 2006 to November 2016 [1]. According to the Köppen classification, the climate is cold semi-arid (BSk), with a mean annual temperature of 14.6 °C and a mean annual precipitation of 373 mm. The soil is mapped as Haplic Calcisol [2].

The field experiment consisted of a 10-year crop rotation, with or without a winter CC between consecutive main summer crops. A maize field planted in 2006 was divided into twelve plots (12 × 12 m^2^) randomly distributed in four replicates of three treatments: barley (*Hordeum vulgare* L.) and vetch (*Vicia villosa* L. or V. *sativa* L.) as CC during the fall and winter period and bare fallow as the control. The main crops (*Zea mays* L. or *Helianthus annuus*) were sown during April and harvested by the end of September.

### 2.2 Sampling and field measurement

The cumulative biomass produced was calculated by adding the aboveground biomass of main crops at harvest and that of the CC at termination from each year. Weed biomass in the fallow treatment was also added in the years when it was relevant (7 out of 10). The annual C input was determined by multiplying the dry biomass remaining in each plot by its C concentration, assuming that all CC residues remained in the field, whereas most of the maize and sunflower biomass was removed from the experiment, leaving the same residue amount (≈ 1000 kg ha^−1^) in all plots. The cumulative C input was obtained by adding up the annual inputs for each plot. The cumulative N fixed by the vetch was determined by adding the N_2_ fixed each year, which was the fixed atmospheric N_2_ calculated by comparing the natural 15 N abundance in vetch and barley plants for each plot [3]. The N content in the CC was obtained by multiplying their dry aboveground biomass by their N concentration for each plot and year.

Soil samples were collected after harvesting the main crop every two years from October 2006 to November 2016. Three soil cores from each plot were collected from 0–5 cm and 5–20 cm depths. These samples were used to determine SOC, SON, WSA and POM. In addition, during the first (2006) and last (2016) years of the field experiment, four soil samples were taken from each treatment with an helicoidal auger (4.5 cm i.d.) and compiled by plot before SOC was analyzed at different depths (0–5,5–20, 20–40, 60–80 and 80–100 cm). The soil C and N retention rates were calculated by adjusting a linear model to SOC and SON with time in each plot, and the tillage effect was removed by subtracting the fallow from the CC treatments.

Soil infiltration was measured directly in the field by a single ring (0.15 m diameter) in 2010 and 2012 and by a double ring (0.15 m inner ring diameter and 0.6 m outer) infiltrometer in 2016 [4]. The water level in the rings was kept at ≈ 5 cm. Four repetitions of the measurement were taken in each plot. The infiltration rate was monitored in each ring every 5 min and was assumed to be stable when it reached a constant flow (i.e., no differences between four consecutive measurements).

At the end of the experiment, a trench (1 m wide, 4 m long and 3 m deep) was dug in the centre of each of the fallow and barley plots to obtain a profile of inorganic N distribution according to depth. Four soil samples were collected along the trench in different intervals down to 3 m and two soil samples were collected with an helicoidal auger (4.5 cm i.d.) down to 4 m. Soil samples were compiled for each layer and plot, extracted with 1 M KCl and analyzed for NH_4_^+^ and NO_3_^−^.

### 2.3 Soil and plant analyses

Soil organic carbon was determined by Walkley–Black method [5]. The C and N concentrations in plant and soil subsamples were determined by the Dumas combustion method (TruMac CN, Leco Instruments, St. Joseph, USA). Vetch and barley samples were analyzed for 15 N concentration with an IRMS Delta Plus XL mass spectrometer (DeltaPlus XL, Thermo Fisher Scientific, Waltham, MA, USA) to calculate the atmospheric N_2_ fixed by the vetch. Water-stable aggregates were determined by wet-sieving of air-dried 1–2 mm aggregates [6], and POM was measured according to Cambardella and Elliott [7]. Soil extracts were analyzed for nitrate and ammonium by spectrophotometry [8].
