# Supplementary material for: Data supporting the cover crops benefits related to soil functionality in a 10-year cropping system
Source: Data Brief. 2018 Apr 18;18:1327–33. doi: 10.1016/j.dib.2018.04.029 (PMC5997093; doi:10.1016/j.dib.2018.04.029)
Supplement: Supplementary file 1 — Supplementary material [file mmc1.docx]

**Conflict of interest**

The authors declared that there is not conflict of interest.
